# Endothelial cell dysfunction: a key determinant for the outcome of allogeneic stem cell transplantation

**DOI:** 10.1038/s41409-021-01390-y

**Published:** 2021-07-12

**Authors:** Thomas Luft, Peter Dreger, Aleksandar Radujkovic

**Affiliations:** Department Medicine V, University of Heidelberg, Heidelberg, Germany

**Keywords:** Medical research, Biological sciences

## Abstract

Allogeneic hematopoietic stem cell transplantation (alloSCT) carries the promise of cure for many malignant and non-malignant diseases of the lympho-hematopoietic system. Although outcome has improved considerably since the pioneering Seattle achievements more than 5 decades ago, non-relapse mortality (NRM) remains a major burden of alloSCT. There is increasing evidence that endothelial dysfunction is involved in many of the life-threatening complications of alloSCT, such as sinusoidal obstruction syndrome/venoocclusive disease, transplant-associated thrombotic microangiopathy, and refractory acute graft-versus host disease. This review delineates the role of the endothelium in severe complications after alloSCT and describes the current status of search for biomarkers predicting endothelial complications, including markers of endothelial vulnerability and markers of endothelial injury. Finally, implications of our current understanding of transplant-associated endothelial pathology for prevention and management of complications after alloSCT are discussed.

## The endothelium—a hinge between extrinsic and intrinsic noxae and post-transplant complications

The endothelium is a semipermeable monolayer of endothelial cells (EC) organized as a complex biological interface that separates all tissues from circulating blood. The vascular endothelium is a highly active organ involved in the regulation of the vascular tone, cellular adhesion and migration, coagulation, vessel wall permeability, and various inflammatory processes [1, 2]. In the setting of alloSCT, host ECs may also participate in adaptive immune responses [3].

**Fig. 1:**
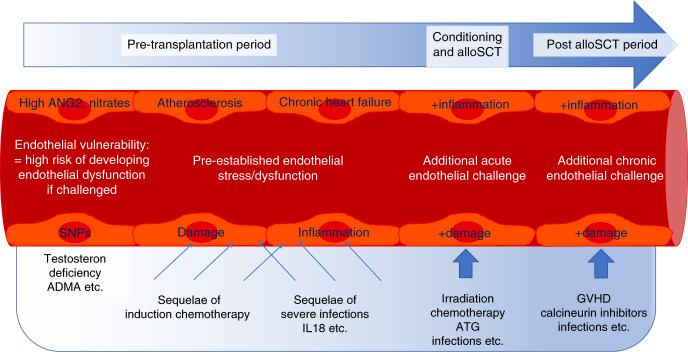
Endothelial challenges during allogeneic stem cell transplantation. Endothelial cells experience stressing influences before, during, and after alloSCT. In the pre-transplantation period, patient-specific endothelial vulnerability and pre-established endothelial damage set the stage for the subsequent challenges during conditioning therapy, immune suppression, and post-transplant complications.

During alloSCT, ECs are consecutively challenged by toxicities of the conditioning regimen and the drugs used for immunosuppressive prophylaxis, inflammatory molecules released by damaged cells and tissues, endotoxins due to damaged mucosal barriers, donor leukocyte engraftment, and alloreactive immune responses [3]. Individual responses of patients’ ECs may be driven by both acquired endothelial distress (caused by comorbidities, pretreatment toxicity, etc.) and an intrinsic endothelial vulnerability (e.g. genetic polymorphisms [4]) (Fig. 1). Possible consequences are EC activation and injury that may progress to an irreversible state of endothelial dysfunction. In turn, a pro-inflammatory, pro-coagulant and pro-apoptotic process is triggered, manifesting as endothelial injury syndromes. Here we focus on the most prominent of them, namely sinusoidal obstruction syndrome/venoocclusive disease (SOS/VOD), transplant-associated thrombotic microangiopathy (TA-TMA), and refractory acute GVHD. Additional endothelial injury syndromes after alloSCT not addressed in this overview include i.a. vascular type idiopathic pneumonia syndrome [5], early fluid retention [6], early bilirubinaemia [7] posterior reversible encephalopathy syndrome, and several subtypes of chronic GVHD [8].

### Biomarkers of selected endothelial injury syndromes after alloSCT

For delineating the pathogenesis, but also for diagnosis and prediction of the main endothelial injury syndromes VOD/SOS, TA-TMA, and refractory acute graft-versus host disease (GVHD), extensive efforts have been made to identify biomarkers of endothelial damage and dysfunction.

### VOD/SOS

manifests as damage of sinusoidal ECs, resulting in the liver injury of various degrees. It is characterized by rapid weight gain, ascites, painful hepatomegaly, and jaundice [9, 10]. Several groups have focused on VOD/SOS-related endothelial biomarkers as summarized in Table 1 [11–16].

**Table 1:** Select studies on endothelial biomarkers in three endothelial complications after alloSCT.

**Endothelial injury syndrome**	**Biomarker**	**Association direction**	**Specimen**	**Time point**	**Application**	**No. of patients**	**References**
SOS/VOD							
	Soluble TM, P-selectin	Increased	Blood plasma	d0 to d + 52	Prediction	25	Catani et al. 1996 [12]
	Soluble TM	Increased	Blood plasma	day+15	Prediction	45	Testa et al. 1996 [16]
	Soluble TM, PAI-1	Increased	Blood plasma	d + 14	ns	28	Nurnberger et al. 1998 [14]
	VWF	Increased	Blood plasma	day0, day+7, day+14	Prognosis	24	Palomo et al. 2010 [15]
	VWF, soluble TM, soluble ICAM-1	Increased	Blood plasma and serum	day-1, day+7	Prediction^b^	38	Cutler et al. 2010 [13]
	5-marker panel: L-ficolin, HA, soluble ST2, ANG2, VCAM-1	Increased	Blood plasma	Onset of symptoms, d0	Diagnosis, prognosis (3-marker panel)	45, 35^c^	Akil et al. 2015 [11]
TA-TMA							
	VWF	Increased	Blood plasma	After engraftment	ns	66	Holler et al. 1989 [19]
	VWF, t-PA	Increased	Blood plasma	d + 19	ns	25	Seeber et al. 1992 [25]
	VWF	Increased	Blood plasma	d + 50	ns	84	Kalhs et al. 1995 [22]
	VWF, soluble TM	Increased	Blood plasma	Onset of symptoms	Diagnosis	52	Zeigler et al. 1996 [26]
	VWF, soluble TM, t-PA	Increased	Blood plasma	d + 14	Prediction	16	Kanamori et al. 1998 [23]
	CFH autoantibodies	Increased	Blood plasma	After diagnosis	ns	3 a	Jodele et al. 2013 [21]
	Soluble terminal complement complex (C5b-9)	Increased	Blood serum	Onset of symptoms	Diagnosis, prognosis	90^d^	Jodele et al. 2014 [20]
	Soluble ST2	Increased	Blood serum	Pre-transplant	Prediction	771	Zeisbrich et al. 2017 [27]
	Soluble ST2	Increased	Blood plasma and serum	d + 14	Prediction	95^c,d^, 110^c,d^, 107^c,d^	Rotz et al. 2017 [24]
Acute GVHD							
	VWF	Increased	Skin biopsy^e^	Onset of aGVHD	Diagnosis	55	Dumler et al. 1989 [33]
	VWF	Increased	Skin biopsy^e^	Onset of aGVHD	ns	44	Sviland et al. 1991 [45]
	ICAM-1	Increased	Duodenal biopsy	Onset of aGVHD	ns	18	Roy et al. 1993 [42]
	VWF, VCAM-1	Increased	Skin biopsy^e^	Onset of aGVHD	Diagnosis	23	Shen et al. 1994 [44]
	VWF, soluble TM	Increased	Blood plasma	Onset of aGVHD	ns	50	Salat et al. 1997 [43]
	Soluble ICAM-1, E-selectin	Increased	Blood plasma and serum	d + 30	Prediction	49	Matsuda et al. 2001 [37]
	ANG2, EMP	Increased	Blood plasma and serum	d + 28	Prognosis	26	Nomura et al. 2008 [41]
	ANG2	Increased	Blood serum	Pre-transplant, onset of aGVHD	Prognosis	48	Luft et al. 2011 [35]
	Endothelial TM	Decreased	Colon biopsy^f^	Onset of aGVHD	Diagnosis	51	Andrulis et al. 2012 [31]
	ANG2	Increased	Blood serum	Pre-transplant	Prognosis	331	Dietrich et al. 2013 [32]
	Bone marrow microvessel density	Increased	Bone marrow	Onset of aGVHD	ns	26	Medinger et al. 2013 [38]
	Soluble ST2	Increased	Blood plasma	d + 14 and start of treatment	Prognosis	381, 296^c^, 302^c^, 75^c^	Van der Lugt et al. 2013 [47]
	Follistatin, PlGF	Increased	Blood plasma and serum	Onset of aGVHD, d + 28	Prognosis	34, 105^c^, 158^c^, 53^c^	Holtan et al. 2015 [34]
	4-marker panel: ANG2, soluble TM, D-dimer, CRP	Increased	Blood plasma	Onset of aGVHD	Prognosis	188	Tatekawa et al. 2016 [46]
	VWF	Increased	Blood plasma	d + 7	Prediction	44	Mir et al. 2017 [39]
	Soluble ST2, REG3α	Increased	Blood plasma and serum	d + 14	Prognosis^g^	225	Nomura et al. 2017 [40]
	CEC	Increased	Whole blood	Onset of aGVHD	Diagnosis	90	Almici et al. 2017 [30]
	Soluble ST2, REG3α	Increased	Blood plasma and serum	1 week after start of aGVHD treatment	Prognosis	236, 142^c^, 129^c^	Major-Monfried et al. 2018 [36]

*aGVHD* acute graft-versus-host disease, *ANG2* angiopoietin 2, *CEC* circulating endothelial cells, *CFH* complement factor H, *CRP* C-reactive protein, *HA* hyaluronic acid, *EMP* endothelial cell-derived microparticles, *ICAM-1* intercellular adhesion molecule 1, *ns* not specified, *PAI-1* plasminogen activator inhibitor type-1, *PlGF* placental growth factor, *SOS/VOD* sinusoidal obstruction syndrome/venoocclusive disease, *ST2* suppression of tumorigenicity 2, *TA-TMA* transplant-associated thrombotic microangiopathy, *TM* thrombomodulin, *t-PA* tissue-type plasminogen activator, *VCAM-1* vascular cell adhesion molecule-1, *VWF* von Willebrand factor.

^a^In patients receiving sirolimus.

^b^Validation cohort(s).

^c^Pediatric patients.

^d^Patient age 0–30 years.

^e^Perivascular extravasation of VWF.

^f^Loss of endothelial TM expression.

^g^Patients treated with recombinant soluble thrombomodulin.

### TA-TMA

is another endothelial syndrome associated with excess mortality in the early post-transplant period. TA-TMA may affect up to 25-30% allografted patients with up to 90% mortality rates in its most severe forms [17]. TA-TMA is characterized by the endothelial injury resulting in microangiopathic hemolytic anemia, platelet consumption, complement dysregulation, and thrombosis and fibrin deposition in the microcirculation [18].

Endothelial biomarkers for diagnosis or prognostication of TA-TMA are summarized in Table 1 [19–27]. Algorithms were proposed to identify high-risk patients most likely benefitting from targeted treatment interventions such as terminal complement blockade (Table 1). Inamoto et al. [28] introduced the concept of intestinal transplant-associated microangiopathy as a separate entity observed in patients with severe steroid-refractory diarrhea not meeting the clinical criteria of systemic TMA or GVHD.

### Acute GVHD

(aGVHD) is the most critical complication following alloSCT, in particular its steroid-refractory form, and represents one of the major causes of mortality. Its complex pathophysiology involves cytokine dysregulation and sequential activation of T cells and monocytes [29]. Upon damage by the early toxicities (conditioning, calcineurin inhibitors (CNI), donor leukocyte engraftment), the endothelium itself may also become a target for activated allogeneic T cells at the onset of aGVHD [3].

First histological evidence of immunologic vascular injury was retrieved from skin biopsies of patients suffering from cutaneous aGVHD, showing perivascular VWF deposition. In addition, loss of endothelial TM, increased expression of ICAM-1 and VCAM-1, and extravasation of vWF was observed in tissue specimens of patients with aGVHD, pointing to EC involvement (Table 1 [30–47]).

Another blood-derived endothelial biomarker considered for aGVHD is ANG2, both as a single marker [35, 48] and as part of a biomarker panel [46]. ANG2 was shown to be predictive for general endothelial damage-related transplant complications, and particularly for the development of treatment-refractory aGVHD (Table 1). Additional endothelial markers are summarized in Table 1.

Finally, ST2 has become an important focus of biomarker research for aGVHD prediction and prognostication [36, 40, 47, 49]. This marker also associates with TA-TMA [24, 27] and thus underlines the link between microangiopathy and lethal complications (Table 1).

## Functional heterogeneity of systemic endothelial cell dysfunction—implications for biomarker studies

Functional heterogeneity is a hallmark of the endothelial cell system [50, 51]. The hypothetical functional definition of ECs as input-output devices [50, 52] emphasizes their role as direct responders to a variety of challenges such as blood pressure, temperature, pH and oxygen pressure, and serum factors [51, 53]. Maintaining homeostasis of tissue perfusion is an important function of ECs that are continuously exposed to stimuli provided by the alternative complement pathway, coagulation factors, cytokines, activated platelets, leukocytes, and occasional infectious agents. Due to their distribution over space and time, hardly two ECs will be exposed to the same set of input signals [54]. Moreover, stochastic or inheritable heterogeneous DNA methylation patterns add to the functional variability of seemingly “homogeneous” mature EC populations [55, 56]. Therefore, tissue-specific stress responses of ECs can explain that even during *systemic* EC dysfunction (e.g. due to CNI, viruses, irradiation etc.), microangiopathy develops *locally* in individual patients [54].

This functional heterogeneity has to be considered in all attempts to define clinical diagnostic criteria for endothelial complications after alloSCT. It also explains the difficulties in defining unequivocal diagnostic criteria for TA-TMA and VOD/SOS. E.g. for TA-TMA, based on expert opinion rather than biology, diverse consensus-defined cut-offs for creatinine, lactate dehydrogenase (LDH), platelet and schistocyte counts, weight gain, and bilirubin levels exist, resulting in discordant diagnostic systems with strongly diverging prognostic impact [20, 57–59].

Ideal markers should be capable of predicting endothelial dysfunction in different clinical settings. Not surprisingly, many endothelial biomarkers described in alloSCT, e.g. ST2 [60], ANG2 [61, 62], ADMA [63], Nitrates [64], TM [65], and others, also predict outcome in cardiovascular disorders. However, these markers were developed generally for diagnosis and/or prognostication of manifest clinical problems rather than for predicting systemic endothelial dysfunction as defined above. In contrast, the goal should be to find biomarkers indicating an increased endothelial risk even before the onset of endothelial complications, ideally before transplant. This risk could consist in a pre-existing subclinical or clinical endothelial defect conferring a generally increased likelihood of endothelial complications and mortality: *endothelial injury*. Alternatively, the risk could consist in the predisposition of an otherwise intact endothelium of becoming dysfunctional only after a triggering event such as GVHD or other second hits: *endothelial vulnerability* (Fig. 1).

### Endothelial vulnerability and endothelial injury

In contrast to the immune system, which is usually completely replaced by cells of donor origin after alloSCT, ECs remain exclusively recipient-derived. This implies that pre-existing defects may impair post-transplant homeostasis of the endothelium and its capacity of enduring eventual challenges, such as the aforementioned noxae, but also the interaction between recipient ECs and donor immune cells and platelets.

**Fig. 2:**
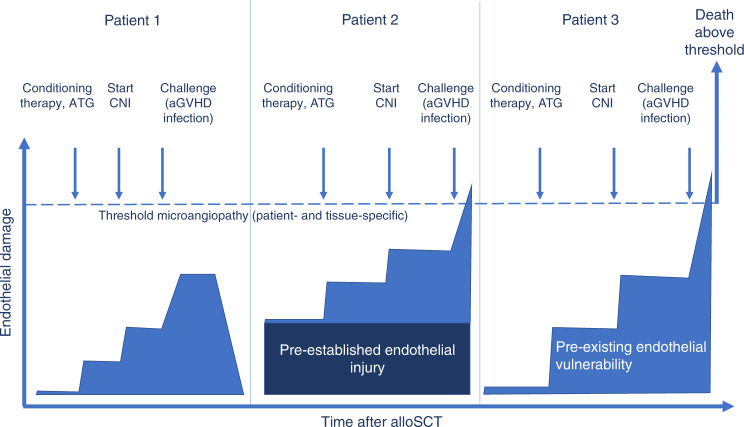
Hypothetical link between pre-established endothelial cell injury and endothelial vulnerability with mortality after alloSCT. Conditioning therapy, immunosuppressive drugs, and post-transplant complications increase endothelial cell distress. In most patients, the threshold to substantial endothelial dysfunction, disturbed microcirculation/microangiopathy and death will not be trespassed (patient 1). Patient 2 with pre-established endothelial cell injury responds similarly to the additional endothelial strains in the context of alloSCT. However, the threshold will be reached due to a lower area of resilience. Patient 3 without pre-established endothelial injury responds more vigorously to the same endothelial challenges due to a patient-specific endothelial vulnerability. The net effect is again an infringement of the threshold and severe complications/death.

The concept of *endothelial vulnerability* is based on the observation that serum markers of endothelial cell distress such as nitrates, ANG2, and ADMA, when elevated before conditioning therapy, are associated with increased NRM only in case of a second hit, namely aGVHD, but not in patients who do not develop aGVHD [32, 35]. Similarly, defined single nucleotide polymorphisms in *recipient* genes related to endothelial integrity, such as the TM and CD40Ligand genes, result in a significantly poorer outcome of patients with aGVHD, without affecting survival in the absence of GVHD [4, 66] (Table 2). This resembles other endothelial complications outside the transplant setting, such as atypical hemolytic-uremic syndromes (aHUS), that are based on pathogenic alterations in various components of the complement pathway but require additional challenges before actual endothelial cell dysfunction occurs [67]. This 2-step model of endothelial vulnerability is also supported by the observation that only a minority of patients with gastrointestinal GVHD shows loss of endothelial surface expression of TM as a sign of endothelial damage at disease onset, although serum CD141 levels subsequently increase in most refractory patients [31, 35]. This supports the view that pre-existing endothelial vulnerability paves the way for a progressive microangiopathy developing under the stress posed by aGVHD, adding to organ damage and finally resulting in steroid refractoriness (Fig. 2) [35].

**Table 2:** Characteristics of endothelial vulnerability and endothelial injury.

**Endothelial vulnerability**	**Endothelial injury**
No impact on outcome without aGVHD	Predicts NRM with and without aGVHD
SEP normalizes risk of NRM	No impact of SEP on NRM
ANG2, nitrates, ADMA, SNPs in THBD and CD40L	EASIX, IL18, testosterone deficiency (men)

*aGVHD* acute graft-versus-host disease, *SEP* statin-based endothelial protection, *NRM* non-relapse mortality, *ANG2* angiopoietin-2, *ADMA* asymmetric dimethyl arginine, *SNPs* single nucleotide polymorphisms, *THBD* thrombomodulin, *EASIX* endothelial activation and stress index, *IL18* interleukin-18.

The lack of predictive power in patients without aGVHD is a strong hint that endothelial vulnerability markers do not reflect manifest endothelial damage. In contrast, endothelial markers measured pre-transplant or early post-transplant which are associated with an increased risk of NRM (or TMA as the common clinical end stage of endothelial damage) independent of GVHD can be considered as indicators of an *actually injured endothelium* associated with already manifest endothelial dysfunction. Examples for this type of markers are ST2 and IL18 [27, 68] (Fig. 1**/**Fig. 2**/**Table 2).

Because the triad of increased creatinine and LDH together with low platelet counts represents the cornerstone of TA-TMA diagnosis, we explored if the ratio of LDH*creatinine/platelets as a continuous, quantitative read-out, termed “Endothelial activation and Stress Index” or *EASIX*, could serve as easy-to-assess surrogate for measuring endothelial injury. Indeed, EASIX measured pre-transplant (EASIX-pre) predicts TA-TMA and NRM, EASIX measured on day 0 of transplantation (EASIX-d0) predicts SOS/VOD, and EASIX measured at the onset of acute GVHD (EASIX-aGVHD) predicts NRM [69–72]. In addition, EASIX-pre and EASIX-d0 correlate with newly recognized syndromes of endothelial cell dysfunction such as early fluid retention [6] and early hyperbilirubinaemia (irrespective of SOS/VOD) [7].

Similar to established endothelial dysfunction markers, EASIX-pre predicts outcome also in patients without aGVHD [71, 72]. Accordingly, EASIX-pre correlates with markers of endothelial injuries, such as IL18, and low IGF1, but not with vulnerability markers [71] (Table 2). In conclusion, EASIX is a readily available indicator of actual endothelial dysfunction throughout the whole peritransplant period. Its applicability in non-transplant clinical settings, such as prognostication of lower risk myelodysplastic syndromes [73], multiple myeloma [74], CAR-T cell therapy, chronic heart disease, and COVID-19 is currently being explored.

### Management implications

Regarding therapeutic interventions for endothelial damage, defibrotide is currently the only approved drug for the treatment of hepatic SOS/VOD. In a historically controlled multicenter open-label phase-III study [75] a significantly better day +100 survival following alloSCT was observed in the defibrotide arm (38%) compared to controls (25%). Results from a compassionate-use [76] and expanded-access treatment program [77] could further verify the efficacy and safety of defibrotide for the treatment of post-transplant SOS/VOD. For a more detailed review on defibrotide treatment and other recent advances in the therapy of endothelial syndromes see references 78–80.

Obviously, the hen-and-egg dilemma—namely whether endothelial defects are cause or consequence of transplant-related complications—is still not completely solved. However, the evidence outlined in the previous section suggests that a pre-existing endothelial aberration is often present, either latent as vulnerability, or as manifest injury (Fig. 1). If this conclusion is correct, in addition to therapeutic strategies, prophylactic or pre-emptive measures for endothelium protection at least in those patients who have a biomarker profile suspicious of endothelial vulnerability are highly warranted.

EC-protective drugs that have been explored include statins, ursodeoxycholic acid (UDA), and defibrotide [4, 27, 69, 81–89]. Regarding statins, preliminary evidence suggests that statin prophylaxis with or without UDA is safe and can reduce the risk of TA-TMA, SOS/VOD, and refractory aGVHD, thereby decreasing NRM (Table 3). Prophylactic UDA reduced NRM and severe aGVHD in a prospective randomized study [89]. Defibrotide prophylaxis reduced the risk of SOS/VOD and aGVHD in a large pediatric prospective randomized trial with a favorable safety profile [86, 90]. Although conclusive rating of the evidence provided by these studies is hampered by considerable heterogeneity of study design, endpoints, and often small sample size, the bottom-line is that there appear to be some efficacy signals for endothelium protection by all three drugs.

**Table 3:** Select studies on intended or incidental prophylaxis with endothelium-protective drugs in allo-SCT.

**Drug**	**Study type and objective (application)**	**Number of patients**	**Target population**	**Outcomes**	**References**
Miscellaneous statins	Retrospective; post-transplant hyperlipidaemia(incidental)	No statins: 541, statins: 220	recipients RD, UD	Grade II-IV aGVHD significantly increased in patients with hiperlipidaemia; statins reduced hyperlidpidaemia without significant side effects	Blaser et al. 2012 [81]
Miscellaneous statins	Retrospective; incidence and severity of aGVHD (incidental)	No statins: 57, statins: 10	Recipients (AML, ALL)	Trend to less aGVHD (II-IV) in the statin group (*p* = 0.08), no effect on cGVHD, no effect on GVL	Hamadani et al. 2008 [83]
Atorvastatin	Prospective single arm; safety, grade II-IV aGVHD (intended)	Statins: 69; 30 (MRD) 39 (MUD)	Recipients	No negative safety signals; preliminary positive efficacy signals.	Kanate et al. 2017 [85]
Miscellaneous statins	Retrospective; GVHD risk(incidental)	No statins: 464, statins: 75	Donors and/or recipients, RD	Grade III-IV aGVHD significantly reduced with donor statin treatment; trend for less NRM with recipient statin treatment; effects seen only with CSA	Rotta et al. 2010 [87]
Miscellaneous statins	Retrospective; GVHD risk, NRM, Relapse, mortality(incidental)	No statins: 1130; statins: 76	Recipients, RD and UD	Chronic GVHD significantly reduced but relapse risk increased with statins;effects seen only with CSA;no statin effect on any other endpoint	Rotta et al. 2010 [88]
Atorvastatin	Prospective single arm; safety, grade II-IV aGVHD (prophylactic use)	Statins: 30	Donors and recipients, RD	No negative safety signals; preliminary positive efficacy signals.	Hamadani et al. 2013 [84]
UDA	Prospective randomized; chronic GVHD and survival outcomes (intended)	No UDA: 119 UDA: 123	Recipients, RD and UD	Grade III-IV aGVHD significantly reduced and NRM and OS significantly improved with UDA; no significant effects on chronic GVHD and relapse risk	Ruutu et al. 2014 [89]
Pravastatin ± UDA	Retrospective cohort comparison; TA-TMA, refractory aGVHD (intended)	No statins/UDA: 356 statins/UDA: 415	Recipients, RD and UD	TA-TMA, refractory aGVHD significantly reduced with statins/UDA	Zeisbrich et al. 2017 [27]
Pravastatin ± UDA	Retrospective cohort comparison; SOS/VOD (intended)	No statins/UDA: 826, statins/UDA: 359	Recipients, RD and UD	SOS/VOD significantly reduced with statins/UDA; effect most pronounced in the highest EASIX quartile	Jiang et al.2020 [69]
Pravastatin ± UDA	Retrospective cohort comparison; survival outcomes (intended)	No statins/UDA: 576 statins/UDA: 344	Recipients, RD and UD	NRM reduced with statins/UDA	Rachakonda et al. 2018 [4]
Defibrotide ± UDA	Prospective randomized; SOS/VOD (intended)	Defibrotide: 180, No defibrotide: 176	Recipients, autologous and allogeneic, pediatric only, high risk	SOS/VOD reduced with defibrotide; grade I-IV aGVHD significantly reduced with defibrotide; no negative safety signals including bleeding events; no effect on TA-TMA, NRM, and overall mortality	Corbacioglu et al. 2012 [82]
Defibrotide + UDA	Retrospective; SOS/VOD (intended)	Defibrotide: 63	Recipients(adult, high risk)	No negative safety signals except for bleeding events in 22%; preliminary positive efficacy signals	Picod et al. 2018 [86]

*aGVHD* acute graft-versus-host disease, *cGVHD* chronic graft-versus-host disease, *GVL* graft-versus-leukemia, *NRM* non-relapse mortality, *RD* related donor, *SOS/VOD* sinusoidal obstruction syndrome/venoocclusive disease, *TA-TMA*, transplant-associated thrombotic microangiopathy, *UD* unrelated donor, *UDA* ursodeoxycholic acid.

To this end, in 2010 we introduced a statin-based endothelial protection (SEP) combining UDA and pravastatin as institutional routine policy for all patients admitted for alloSCT. This was associated with attenuation of excess NRM in aGVHD patients with biomarkers of endothelial vulnerability, whereas NRM in patients without evidence for endothelial vulnerability remained unchanged (Table 3). Notably, incidences of SOS/VOD and TA-TMA were also reduced in patients with SEP, as compared to (non-randomized) controls [27, 69, 71].

Thus, although randomized studies are missing, the combination of statins and UDA appears to have the capacity to alleviate the complications linked to endothelial vulnerability, but not to repair manifest severe endothelial injury (Table 2). Therefore, new approaches are necessary for the prevention and treatment of endothelial dysfunction caused by pre-existing endothelial damage. Two obvious principle strategies (which are not mutually exclusive) for achieving this will be detailed in the following: a) re-shaping anti-neoplastic and immunosuppressive regimens in order to preserve endothelial integrity; and b) exploring novel agents for endothelial protection or repair for patients with established endothelial dysfunction.

## Adjusting anti-neoplastic and immunosuppressive regimens to endothelial cell function

Here, two basic questions have to be addressed: first, where does the pre-existing endothelial lesion derive from - and second, to which extent do conditioning regimens, blood pressure medications, antibiotic, antiviral and antifungal drugs, and immunosuppression add to impairment of physical and functional endothelial integrity in individual patients?

The first question requires a thorough work-up of the endothelial toxicity of agents commonly used in the pre-SCT setting, such as fludarabine, alkylators, and anthracyclines, but also irradiation [91, 92], but of course also of the contributions of vascular comorbidity unrelated to the neoplastic disease. Regarding the second question, a large variety of drugs frequently employed during or after SCT, such as CNI, sirolimus, calcium channel blockers, and angiotensin-II inhibitors can affect endothelial integrity and may demand patient-based endothelial monitoring [93–96]. Because of its easy accessibility, also for retrospective analyses, EASIX might be a particularly useful tool for this purpose.

## How to explore novel endothelial protective agents

There is a paucity of agents with the capacity of protecting or restoring EC integrity. Given the potential side effects of novel drugs being explored for this purpose, high-risk populations who are most in need of such medications need to be identified. Our experiences with SEP show that endothelial protection may differ for endothelial vulnerability settings and manifest endothelial cell injury. Similarly, the reported benefits of defibrotide and—in children—C5 inhibitors (e.g. Eculizumab [97]) will have to be analyzed for differential efficacy in patients with different endothelial risk. In adult patients, serious toxicities, e.g. fatal infections with complement C5 inhibitors [98, 99], strongly discourage using this approach outside of clinical trials.

In addition to searching for novel EC-promoting agents, we should also consider incorporating the established knowledge of cardiovascular medicine for endothelial protection, e.g. by investigating pre-emptive use of statins, beta-adrenergic antagonists, angiotensin-converting enzyme inhibitors, acetyl salicylate, or N-acetylcystein [100] amongst others, in patients with evidence for endothelial injury. The EASIX toolkit seems to be particularly practical for classifying endothelial risk for this purpose.

## Conclusion

EC dysfunction syndromes are increasingly recognized as important contributors to mortality and morbidity after alloSCT. Although their pathogenesis is not uniform and the overwhelming functional heterogeneity of EC can channel systemic endothelial disorders into tissue-specific, local microangiopathies, the common final path of EC dysfunction syndromes is a severe and mostly irreversible alteration of EC integrity. There is growing evidence that the manifestation of clinically effective EC disintegration is at least partially driven by distinct pre-existing endothelial defects which can be defined as endothelial vulnerability and endothelial injury, respectively. Whereas endothelial injury represents manifest lesions resulting in permanent endothelial dysfunction, endothelial vulnerability describes latent defects translating into endothelial dysfunction only upon a second hit. These two conditions can be distinguished by biomarker profiling with a prominent role for EASIX. While endothelial vulnerability might potentially be overcome by prophylactic use of endothelium-protective drugs, such as statins and UDA, effective tools for treating manifest endothelial damage—except for defibrotide in specific settings—are missing. Thus, novel approaches to target endothelial injury and its devastating clinical sequelae appear to be a high-priority goal in order to reduce the risks of allo-SCT.

## References

[1] Deanfield JE, Halcox JP, Rabelink TJ (2007). Endothelial function and dysfunction: testing and clinical relevance. Circulation.

[2] Pober JS, Sessa WC (2007). Evolving functions of endothelial cells in inflammation. Nat Rev Immunol.

[3] Biedermann BC (2008). Vascular endothelium and graft-versus-host disease. Best Pr Res Clin Haematol.

[4] Rachakonda SP, Dai H, Penack O, Blau O, Blau IW, Radujkovic A (2018). Single nucleotide polymorphisms in CD40L predict endothelial complications and mortality after allogeneic stem-cell transplantation. J Clin Oncol.

[5] Wenger DS, Triplette M, Crothers K, Cheng GS, Hill JA, Milano F (2020). Incidence, risk factors, and outcomes of idiopathic pneumonia syndrome after allogeneic hematopoietic cell transplantation. Biol Blood Marrow Transplant.

[6] Varma A, Rondon G, Srour SA, Chen J, Ledesma C, Champlin RE (2020). Endothelial activation and stress index (EASIX) at admission predicts fluid overload in recipients of allogeneic stem cell transplantation. Biol Blood Marrow Transplant.

[7] Dai H, Penack O, Radujkovic A, Schult D, Majer-Lauterbach J, Blau IW, et al. Early Bilirubinemia after allogeneic stem cell transplantation—an endothelial complication. Bone Marrow Transplant. 2021. 10.1038/s41409-020-01186-6. 10.1038/s41409-020-01186-6 PMC8263345 33517355

[8] Biedermann BC, Sahner S, Gregor M, Tsakiris DA, Jeanneret C, Pober JS (2002). Endothelial injury mediated by cytotoxic T lymphocytes and loss of microvessels in chronic graft versus host disease. Lancet.

[9] Mohty M, Malard F, Abecassis M, Aerts E, Alaskar AS, Aljurf M (2015). Sinusoidal obstruction syndrome/veno-occlusive disease: current situation and perspectives-a position statement from the European Society for Blood and Marrow Transplantation (EBMT). Bone Marrow Transplant.

[10] Mohty M, Malard F, Abecassis M, Aerts E, Alaskar AS, Aljurf M (2016). Revised diagnosis and severity criteria for sinusoidal obstruction syndrome/veno-occlusive disease in adult patients: a new classification from the European Society for Blood and Marrow Transplantation. Bone Marrow Transplant.

[11] Akil A, Zhang Q, Mumaw CL, Raiker N, Yu J, Velez de Mendizabal N (2015). Biomarkers for Diagnosis and prognosis of sinusoidal obstruction syndrome after hematopoietic cell transplantation. Biol Blood Marrow Transplant.

[12] Catani L, Gugliotta L, Vianelli N, Nocentini F, Baravelli S, Bandini G (1996). Endothelium and bone marrow transplantation. Bone Marrow Transplant.

[13] Cutler C, Kim HT, Ayanian S, Bradwin G, Revta C, Aldridge J (2010). Prediction of veno-occlusive disease using biomarkers of endothelial injury. Biol Blood Marrow Transplant.

[14] Nurnberger W, Michelmann I, Burdach S, Gobel U (1998). Endothelial dysfunction after bone marrow transplantation: increase of soluble thrombomodulin and PAI-1 in patients with multiple transplant-related complications. Ann Hematol.

[15] Palomo M, Diaz-Ricart M, Carbo C, Rovira M, Fernandez-Aviles F, Escolar G (2009). The release of soluble factors contributing to endothelial activation and damage after hematopoietic stem cell transplantation is not limited to the allogeneic setting and involves several pathogenic mechanisms. Biol Blood Marrow Transplant.

[16] Testa S, Manna A, Porcellini A, Maffi F, Morstabilini G, Denti N (1996). Increased plasma level of vascular endothelial glycoprotein thrombomodulin as an early indicator of endothelial damage in bone marrow transplantation. Bone Marrow Transplant.

[17] Laskin BL, Goebel J, Davies SM, Jodele S (2011). Small vessels, big trouble in the kidneys and beyond: hematopoietic stem cell transplantation-associated thrombotic microangiopathy. Blood.

[18] Daly AS, Xenocostas A, Lipton JH (2002). Transplantation-associated thrombotic microangiopathy: twenty-two years later. Bone Marrow Transplant.

[19] Holler E, Kolb HJ, Hiller E, Mraz W, Lehmacher W, Gleixner B (1989). Microangiopathy in patients on cyclosporine prophylaxis who developed acute graft-versus-host disease after HLA-identical bone marrow transplantation. Blood.

[20] Jodele S, Laskin BL, Dandoy CE, Myers KC, El-Bietar J, Davies SM, et al. A new paradigm: Diagnosis and management of HSCT-associated thrombotic microangiopathy as multi-system endothelial injury. Blood Rev. 2014. 10.1016/j.blre.2014.11.001 10.1016/j.blre.2014.11.001 PMC4659438 25483393

[21] Jodele S, Licht C, Goebel J, Dixon BP, Zhang K, Sivakumaran TA (2013). Abnormalities in the alternative pathway of complement in children with hematopoietic stem cell transplant-associated thrombotic microangiopathy. Blood.

[22] Kalhs P, Brugger S, Schwarzinger I, Greinix HT, Keil F, Kyrle PA (1995). Microangiopathy following allogeneic marrow transplantation. Association with cyclosporine and methylprednisolone for graft-versus-host disease prophylaxis. Transplantation.

[23] Kanamori H, Maruta A, Sasaki S, Yamazaki E, Ueda S, Katoh K (1998). Diagnostic value of hemostatic parameters in bone marrow transplant-associated thrombotic microangiopathy. Bone Marrow Transplant.

[24] Rotz SJ, Dandoy CE, Davies SM (2017). ST2 and Endothelial Injury as a Link between GVHD and Microangiopathy. N Engl J Med.

[25] Seeber C, Hiller E, Holler E, Kolb HJ (1992). Increased levels of tissue plasminogen activator (t-PA) and tissue plasminogen activator inhibitor (PAI) correlate with tumor necrosis factor alpha (TNF alpha)-release in patients suffering from microangiopathy following allogeneic bone marrow transplantation (BMT). Thromb Res.

[26] Zeigler ZR, Rosenfeld CS, Andrews DF, Nemunaitis J, Raymond JM, Shadduck RK (1996). Plasma von Willebrand Factor Antigen (vWF:AG) and thrombomodulin (TM) levels in adult thrombotic thrombocytopenic purpura/hemolytic uremic syndromes (TTP/HUS) and bone marrow transplant-associated thrombotic microangiopathy (BMT-TM). Am J Hematol.

[27] Zeisbrich M, Becker N, Benner A, Radujkovic A, Schmitt K, Beimler J, et al. Transplant-associated thrombotic microangiopathy is an endothelial complication associated with refractoriness of acute GvHD. Bone Marrow Transplant. 2017. 10.1038/bmt.2017.119 10.1038/bmt.2017.119 28650448

[28] Inamoto Y, Ito M, Suzuki R, Nishida T, Iida H, Kohno A (2009). Clinicopathological manifestations and treatment of intestinal transplant-associated microangiopathy. Bone Marrow Transplant.

[29] Zeiser R, Blazar BR, Acute Graft-versus-Host Disease - (2017). Biologic process, prevention, and therapy. N Engl J Med.

[30] Almici C, Skert C, Bruno B, Bianchetti A, Verardi R, Di Palma A (2017). Circulating endothelial cell count: a reliable marker of endothelial damage in patients undergoing hematopoietic stem cell transplantation. Bone Marrow Transplant.

[31] Andrulis M, Dietrich S, Longerich T, Koschny R, Burian M, Schmitt-Gräf A (2012). Loss of endothelial thrombomodulin predicts response to steroid therapy and survival in acute intestinal graft-versus-host disease. Haematologica.

[32] Dietrich S, Falk CS, Benner A, Karamustafa S, Hahn E, Andrulis M (2013). Endothelial vulnerability and endothelial damage are associated with risk of graft-versus-host disease and response to steroid treatment. Biol Blood Marrow Transplant.

[33] Dumler JS, Beschorner WE, Farmer ER, Di Gennaro KA, Saral R, Santos GW. Endothelial-cell injury in cutaneous acute graft-versus-host disease. Am J Pathol. 1989;135:1097–103. PMC1880480 2596572

[34] Holtan SG, Verneris MR, Schultz KR, Newell LF, Meyers G, He F (2015). Circulating angiogenic factors associated with response and survival in patients with acute graft-versus-host disease: results from Blood and Marrow Transplant Clinical Trials Network 0302 and 0802. Biol Blood Marrow Transplant.

[35] Maeda T, Wakasawa T, Shima Y, Tsuboi I, Aizawa S, Tamai I (2011). Steroid-refractory GVHD: T-cell attack within a vulnerable endothelial system. Blood.

[36] Major-Monfried H, Renteria AS, Pawarode A, Reddy P, Ayuk F, Holler E (2018). MAGIC biomarkers predict long-term outcomes for steroid-resistant acute GVHD. Blood.

[37] Matsuda Y, Hara J, Osugi Y, Tokimasa S, Fujisaki H, Takai K (2001). Serum levels of soluble adhesion molecules in stem cell transplantation-related complications. Bone Marrow Transplant.

[38] Medinger M, Tichelli A, Bucher C, Halter J, Dirnhofer S, Rovo A (2013). GVHD after allogeneic haematopoietic SCT for AML: angiogenesis, vascular endothelial growth factor and VEGF receptor expression in the BM. Bone Marrow Transplant.

[39] Mir E, Palomo M, Rovira M, Pereira A, Escolar G, Penack O (2017). Endothelial damage is aggravated in acute GvHD and could predict its development. Bone Marrow Transplant.

[40] Nomura S, Ishii K, Fujita S, Nakaya A, Satake A, Ito T (2017). Associations between acute GVHD-related biomarkers and endothelial cell activation after allogeneic hematopoietic stem cell transplantation. Transpl. Immunol.

[41] Nomura S, Ishii K, Inami N, Kimura Y, Uoshima N, Ishida H (2008). Evaluation of angiopoietins and cell-derived microparticles after stem cell transplantation. Biol Blood Marrow Transplant.

[42] Roy J, Platt JL, Weisdorf DJ (1993). The immunopathology of upper gastrointestinal acute graft-versus-host disease. Lymphoid cells endothelial Adhes molecules. Transplantation.

[43] Salat C, Holler E, Kolb HJ, Pihusch R, Reinhardt B, Hiller E (1997). Endothelial cell markers in bone marrow transplant recipients with and without acute graft-versus-host disease. Bone Marrow Transplant.

[44] Shen N, Ffrench P, Guyotat D, Ffrench M, Fiere D, Bryon PA (1994). Expression of adhesion molecules in endothelial cells during allogeneic bone marrow transplantation. Eur J Haematol.

[45] Sviland L, Sale GE, Myerson D (1991). Endothelial changes in cutaneous graft-versus-host disease: a comparison between HLA matched and mismatched recipients of bone marrow transplantation. Bone Marrow Transplant.

[46] Tatekawa S, Kohno A, Ozeki K, Watamoto K, Ueda N, Yamaguchi Y (2016). A novel diagnostic and prognostic biomarker panel for endothelial cell damage-related complications in allogeneic transplantation. Biol Blood Marrow Transplant.

[47] Vander Lugt MT, Braun TM, Hanash S, Ritz J, Ho VT, Antin JH (2013). ST2 as a marker for risk of therapy-resistant graft-versus-host disease and death. N Engl J Med.

[48] Ueda N, Chihara D, Kohno A, Tatekawa S, Ozeki K, Watamoto K (2014). Predictive value of circulating angiopoietin-2 for endothelial damage-related complications in allogeneic hematopoietic stem cell transplantation. Biol Blood Marrow Transplant.

[49] Paczesny S. Post-haematopoietic cell transplantation outcomes: why ST2 became a ‘golden nugget’ biomarker. Br J Haematol. 2020. 10.1111/bjh.16497 10.1111/bjh.16497 PMC7415515 32039480

[50] Aird WC. Endothelium in health and disease. Pharmacol Rep. 2008;60:139–43. 18276995

[51] Kalucka J, de Rooij L, Goveia J, Rohlenova K, Dumas SJ, Meta E (2020). Single-cell transcriptome atlas of murine endothelial cells. Cell.

[52] Regan ER, Aird WC (2012). Dynamical systems approach to endothelial heterogeneity. Circ Res.

[53] Aird WC (2005). Spatial and temporal dynamics of the endothelium. J Thromb Haemost.

[54] Aird WC (2007). Phenotypic heterogeneity of the endothelium: I. Structure, function, and mechanisms. Circ Res.

[55] Turgeon PJ, Chan GC, Chen L, Jamal AN, Yan MS, Ho JJD (2020). Epigenetic Heterogeneity and mitotic heritability prime endothelial cell gene induction. J Immunol.

[56] Yuan L, Chan GC, Beeler D, Janes L, Spokes KC, Dharaneeswaran H (2016). A role of stochastic phenotype switching in generating mosaic endothelial cell heterogeneity. Nat Commun.

[57] Cho BS, Yahng SA, Lee SE, Eom KS, Kim YJ, Kim HJ (2010). Validation of recently proposed consensus criteria for thrombotic microangiopathy after allogeneic hematopoietic stem-cell transplantation. Transplantation.

[58] Ho VT, Cutler C, Carter S, Martin P, Adams R, Horowitz M (2005). Blood and marrow transplant clinical trials network toxicity committee consensus summary: thrombotic microangiopathy after hematopoietic stem cell transplantation. Biol Blood Marrow Transplant.

[59] Ruutu T, Barosi G, Benjamin RJ, Clark RE, George JN, Gratwohl A (2007). Diagnostic criteria for hematopoietic stem cell transplant-associated microangiopathy: results of a consensus process by an International Working Group. Haematologica.

[60] Shimpo M, Morrow DA, Weinberg EO, Sabatine MS, Murphy SA, Antman EM (2004). Serum levels of the interleukin-1 receptor family member ST2 predict mortality and clinical outcome in acute myocardial infarction. Circulation.

[61] Patel JV, Lim HS, Varughese GI, Hughes EA, Lip GY (2008). Angiopoietin-2 levels as a biomarker of cardiovascular risk in patients with hypertension. Ann Med.

[62] Smadja DM, Guerin CL, Chocron R, Yatim N, Boussier J, Gendron N, et al. Angiopoietin-2 as a marker of endothelial activation is a good predictor factor for intensive care unit admission of COVID-19 patients. Angiogenesis. 2020. 10.1007/s10456-020-09730-0 10.1007/s10456-020-09730-0 PMC7250589 32458111

[63] Kielstein JT, Zoccali C (2008). Asymmetric dimethylarginine: a novel marker of risk and a potential target for therapy in chronic kidney disease. Curr Opin Nephrol Hypertens.

[64] Gumanova NG, Deev AD, Zhang W, Kots AY, Shalnova SA (2017). Serum nitrite and nitrate levels, NOx, can predict cardiovascular mortality in the elderly in a 3-year follow-up study. Biofactors.

[65] Wu KK, Aleksic N, Ballantyne CM, Ahn C, Juneja H, Boerwinkle E. Interaction between soluble thrombomodulin and intercellular adhesion molecule-1 in predicting risk of coronary heart disease. Circulation. 2003;107:1729–32. 10.1161/01.CIR.0000064894.97094.4F 12668515

[66] Rachakonda SP, Penack O, Dietrich S, Blau O, Blau IW, Radujkovic A (2014). Single-nucleotide polymorphisms within the thrombomodulin gene (THBD) predict mortality in patients with graft-versus-host disease. J Clin Oncol.

[67] Jokiranta TS HUS and atypical HUS. Blood. 2017;129(21)**:**2847–56. 10.1182/blood-2016-11-709865 10.1182/blood-2016-11-709865 PMC5445567 28416508

[68] Radujkovic A, Kordelas L, Dai H, Schult D, Majer-Lauterbach J, Beelen D (2019). Interleukin-18 and outcome after allogeneic stem cell transplantation: a retrospective cohort study. EBioMedicine.

[69] Jiang S, Penack O, Terzer T, Schult D, Majer-Lauterbach J, Radujkovic A et al. Predicting sinusoidal obstruction syndrome after allogeneic stem cell transplantation with the EASIX biomarker panel. Haematologica. 2020. 10.3324/haematol.2019.238790 10.3324/haematol.2019.238790 PMC7849560 31974195

[70] Luft T, Benner A, Jodele S, Dandoy CE, Storb R, Gooley T (2017). EASIX in patients with acute graft-versus-host disease: a retrospective cohort analysis. Lancet Haematol.

[71] Luft T, Benner A, Terzer T, Jodele S, Dandoy CE, Storb R (2020). EASIX and mortality after allogeneic stem cell transplantation. Bone Marrow Transplant.

[72] Shouval R, Fein JA, Shouval A, Danylesko I, Shem-Tov N, Zlotnik M (2019). External validation and comparison of multiple prognostic scores in allogeneic hematopoietic stem cell transplantation. Blood Adv.

[73] Merz A, Germing U, Kobbe G, Kaivers J, Jauch A, Radujkovic A (2019). EASIX for prediction of survival in lower-risk myelodysplastic syndromes. Blood Cancer J.

[74] Song GY, Jung SH, Kim K, Kim SJ, Yoon SE, Lee HS (2020). Endothelial activation and stress index (EASIX) is a reliable predictor for overall survival in patients with multiple myeloma. BMC Cancer.

[75] Richardson PG, Riches ML, Kernan NA, Brochstein JA, Mineishi S, Termuhlen AM (2016). Phase 3 trial of defibrotide for the treatment of severe veno-occlusive disease and multi-organ failure. Blood.

[76] Corbacioglu S, Carreras E, Mohty M, Pagliuca A, Boelens JJ, Damaj G (2016). Defibrotide for the treatment of hepatic veno-occlusive disease: final results from the International Compassionate-use Program. Biol Blood Marrow Transplant.

[77] Kernan NA, Grupp S, Smith AR, Arai S, Triplett B, Antin JH (2018). Final results from a defibrotide treatment-IND study for patients with hepatic veno-occlusive disease/sinusoidal obstruction syndrome. Br J Haematol.

[78] Jodele S (2018). Complement in pathophysiology and treatment of transplant-associated thrombotic microangiopathies. Semin Hematol.

[79] Penack O, Marchetti M, Ruutu T, Aljurf M, Bacigalupo A, Bonifazi F (2020). Prophylaxis and management of graft versus host disease after stem-cell transplantation for haematological malignancies: updated consensus recommendations of the European Society for Blood and Marrow Transplantation. Lancet Haematol.

[80] Richardson P, Aggarwal S, Topaloglu O, Villa KF, Corbacioglu S (2019). Systematic review of defibrotide studies in the treatment of veno-occlusive disease/sinusoidal obstruction syndrome (VOD/SOS). Bone Marrow Transplant.

[81] Blaser BW, Kim HT, Alyea EP, 3rd, Ho VT, Cutler C, Armand P et al. Hyperlipidemia and statin use after allogeneic hematopoietic stem cell transplantation. Biol Blood Marrow Transplant. 2011. 10.1016/j.bbmt.2011.08.003 10.1016/j.bbmt.2011.08.003 PMC3805283 21839706

[82] Corbacioglu S, Kernan N, Lehmann L, Brochstein J, Revta C, Grupp S (2012). Defibrotide for the treatment of hepatic veno-occlusive disease in children after hematopoietic stem cell transplantation. Expert Rev Hematol.

[83] Hamadani M, Awan FT, Devine SM The impact of HMG-CoA reductase inhibition on the incidence and severity of graft-versus-host disease in patients with acute leukemia undergoing allogeneic transplantation. Blood. 2008;111(7)**:**3901–2. 10.1182/blood-2008-01-132050 10.1182/blood-2008-01-132050 18362217

[84] Hamadani M, Gibson LF, Remick SC, Wen S, Petros W, Tse W (2013). Sibling donor and recipient immune modulation with atorvastatin for the prophylaxis of acute graft-versus-host disease. J Clin Oncol.

[85] Kanate AS, Hari PN, Pasquini MC, Visotcky A, Ahn KW, Boyd J (2017). Recipient immune modulation with atorvastatin for acute graft-versus-host disease prophylaxis after allogeneic transplantation. Biol Blood Marrow Transplant.

[86] Picod A, Bonnin A, Battipaglia G, Giannotti F, Ruggeri A, Brissot E (2018). Defibrotide for sinusoidal obstruction syndrome/veno-occlusive disease prophylaxis in high-risk adult patients: a single-center experience study. Biol Blood Marrow Transplant.

[87] Rotta M, Storer BE, Storb R, Martin PJ, Flowers ME, Vernon MS (2010). Impact of recipient statin treatment on graft-versus-host disease after allogeneic hematopoietic cell transplantation. Biol Blood Marrow Transplant.

[88] Rotta M, Storer BE, Storb RF, Martin PJ, Heimfeld S, Peffer A (2010). Donor statin treatment protects against severe acute graft-versus-host disease after related allogeneic hematopoietic cell transplantation. Blood.

[89] Ruutu T, Juvonen E, Remberger M, Remes K, Volin L, Mattsson J (2014). Improved survival with ursodeoxycholic acid prophylaxis in allogeneic stem cell transplantation: long-term follow-up of a randomized study. Biol Blood Marrow Transplant.

[90] Corbacioglu S, Cesaro S, Faraci M, Valteau-Couanet D, Gruhn B, Rovelli A (2012). Defibrotide for prophylaxis of hepatic veno-occlusive disease in paediatric haemopoietic stem-cell transplantation: an open-label, phase 3, randomised controlled trial. Lancet.

[91] Venkatesulu BP, Sanders KL, Hsieh CE, Kim BK, Krishnan S (2019). Biomarkers of radiation-induced vascular injury. Cancer Rep. (Hoboken).

[92] Wojcik T, Szczesny E, Chlopicki S. Detrimental effects of chemotherapeutics and other drugs on the endothelium: a call for endothelial toxicity profiling. Pharmacol Rep. 2015;67(4):811–7. 10.1016/j.pharep.2015.03.022 26321285

[93] Lewis C, Kim HT, Roeker LE, Cutler C, Koreth J, Nikiforow S (2020). Incidence, predictors, and outcomes of veno-occlusive disease/sinusoidal obstruction syndrome after reduced-intensity allogeneic hematopoietic cell transplantation. Biol Blood Marrow Transplant.

[94] Radenkovic M, Stojanovic M, Prostran M. Calcium channel blockers in restoration of endothelial function: systematic review and meta-analysis of randomized controlled trials. Curr Med Chem. 2019;26(29)**:**5579–95. 10.2174/0929867325666180713144806 30009701

[95] Renner B, Klawitter J, Goldberg R, McCullough JW, Ferreira VP, Cooper JE (2013). Cyclosporine induces endothelial cell release of complement-activating microparticles. J Am Soc Nephrol.

[96] Rodrigues-Diez R, González-Guerrero C, Ocaña-Salceda C, Rodrigues-Diez RR, Egido J, Ortiz A (2016). Calcineurin inhibitors cyclosporine A and tacrolimus induce vascular inflammation and endothelial activation through TLR4 signaling. Sci Rep.

[97] Jodele S, Dandoy CE, Myers KC, El-Bietar J, Nelson A, Wallace G (2016). New approaches in the diagnosis, pathophysiology, and treatment of pediatric hematopoietic stem cell transplantation-associated thrombotic microangiopathy. Transfus Apher Sci.

[98] Bohl SR, Kuchenbauer F, von Harsdorf S, Kloevekorn N, Schönsteiner SS, Rouhi A (2017). Thrombotic microangiopathy after allogeneic stem cell transplantation: a comparison of eculizumab therapy and conventional therapy. Biol Blood Marrow Transplant.

[99] Vasu S, Wu H, Satoskar A, Puto M, Roddy J, Blum W (2016). Eculizumab therapy in adults with allogeneic hematopoietic cell transplant-associated thrombotic microangiopathy. Bone Marrow Transplant.

[100] Kong Y, Wang Y, Zhang YY, Shi MM, Mo XD, Sun YQ (2019). Prophylactic oral NAC reduced poor hematopoietic reconstitution by improving endothelial cells after haploidentical transplantation. Blood Adv.

